# Computer Modeling of Alzheimer’s Disease—Simulations of Synaptic Plasticity and Memory in the CA3-CA1 Hippocampal Formation Microcircuit

**DOI:** 10.3390/molecules24101909

**Published:** 2019-05-17

**Authors:** Dariusz Świetlik, Jacek Białowąs, Janusz Moryś, Ilona Klejbor, Aida Kusiak

**Affiliations:** 1 Intrafaculty College of Medical Informatics and Biostatistics, Medical University of Gdańsk, 1 Debinki St., 80-211 Gdańsk, Poland; 2 Department of Anatomy and Neurobiology, Medical University of Gdańsk, 1 Debinki St., 80-211 Gdańsk, Poland; jacekwb@gumed.edu.pl (J.B.); janusz.morys@gumed.edu.pl (J.M.); ilona.klejbor@gumed.edu.pl (I.K.); 3 Department of Periodontology and Oral Mucosa Diseases, Medical University of Gdańsk, 1a Debowa St., 80-204 Gdańsk, Poland; aida.kusiak@gumed.edu.pl

**Keywords:** Alzheimer’s disease, hippocampus, LTP, theta rhythm, computer simulation

## Abstract

This paper aims to present computer modeling of synaptic plasticity and memory in the CA3-CA1 hippocampal formation microcircuit. The computer simulations showed a comparison of a pathological model in which Alzheimer’s disease (AD) was simulated by synaptic degradation in the hippocampus and control model (healthy) of CA3-CA1 networks with modification of weights for the memory. There were statistically higher spike values of both CA1 and CA3 pyramidal cells in the control model than in the pathological model (p = 0.0042 for CA1 and p = 0.0033 for CA3). A similar outcome was achieved for frequency (p = 0.0002 for CA1 and p = 0.0001 for CA3). The entropy of pyramidal cells of the healthy CA3 network seemed to be significantly higher than that of AD (p = 0.0304). We need to study a lot of physiological parameters and their combinations of the CA3-CA1 hippocampal formation microcircuit to understand AD. High statistically correlations were obtained between memory, spikes and synaptic deletion in both CA1 and CA3 cells.

## 1. Introduction

Neural models can be divided into connectionist or biophysical types, both of which have been included in experiments of brain network simulations conducted to understand the fundamental mechanisms of Alzheimer’s disease (AD). Neurons are used to create models of brain processes connected with associative memory formation caused by the increasing significance of synaptic connections and simple spatiotemporal integration in this phenomenon [1,2,3].

The biophysical neural model exerts the particular biophysical features of a single neuron (noted as a spiking neuron), and these features contain the physiological impact of neuromodulation, including bursting and spiking. Spiking neuron cells are framed based on proper experimental data received in ion channel properties. The ion channels transmit a presynaptic neurotransmitter action into membrane depolarization and repolarization, the consequence of which is the firing of action potentials [4,5,6,7,8]. These models seem to be connected networks of spiking neurons whose role is to simulate the features of individual neurons and associated occurrences. According to Menschik et al. [9], they are biological analogs of the connectionist neural models and could take part in simulations of memory dysfunctions such as AD; however, the use of theta and gamma oscillations, cholinergic neuromodulation, diversity of neuron types and input patterns are required [9,10,11,12]. It is used only to present the maintenance of a small set of neurons. The connectionist neural model is less demanding and needs fewer simulation details than a biophysical one. In general, neural models have been evolved to simulate the functions of the hippocampus with regard to memory formation. It is proven that both of those models clearly present some universal behaviors depending on the manipulations of network factors. In addition to artificial neural networks, there are useful tool simulations of pyramidal cells, CA1 and CA3 of the hippocampus, which support the diagnostics of AD [13,14,15,16].

In the neural networks on the high level of biological realism, the computation with the timing of individual spikes (action potentials) must be used. And after arrival of an action potential at the excitatory synapse we have an embarrassment of riches in which synaptic plasticity does occur. The biological neurons such as pyramidal cells in the hippocampus or cortex show various types of long-term synaptic potentiation (LTP) or depression (LTD) with presumably dendritic locations of their mechanisms throughout the N-methyl-D-aspartate (NMDA) glutamate receptors [17,18,19]. As for computational models of a neuron, since biological plausibility and computational efficiency are contradictory objectives, the possible detailed models of those processes are not suitable for the building of large scale networks [20,21,22]. As such, the most frequently used rule is the spike time dependent plasticity (STDP) being completed with details of intracellular calcium turnover [23,24,25]. STDP allows both the increase and decrement of synaptic weights in dependency of succession of presynaptic and postsynaptic spikes. 

## 2. Materials and Methods

**Figure 1:**
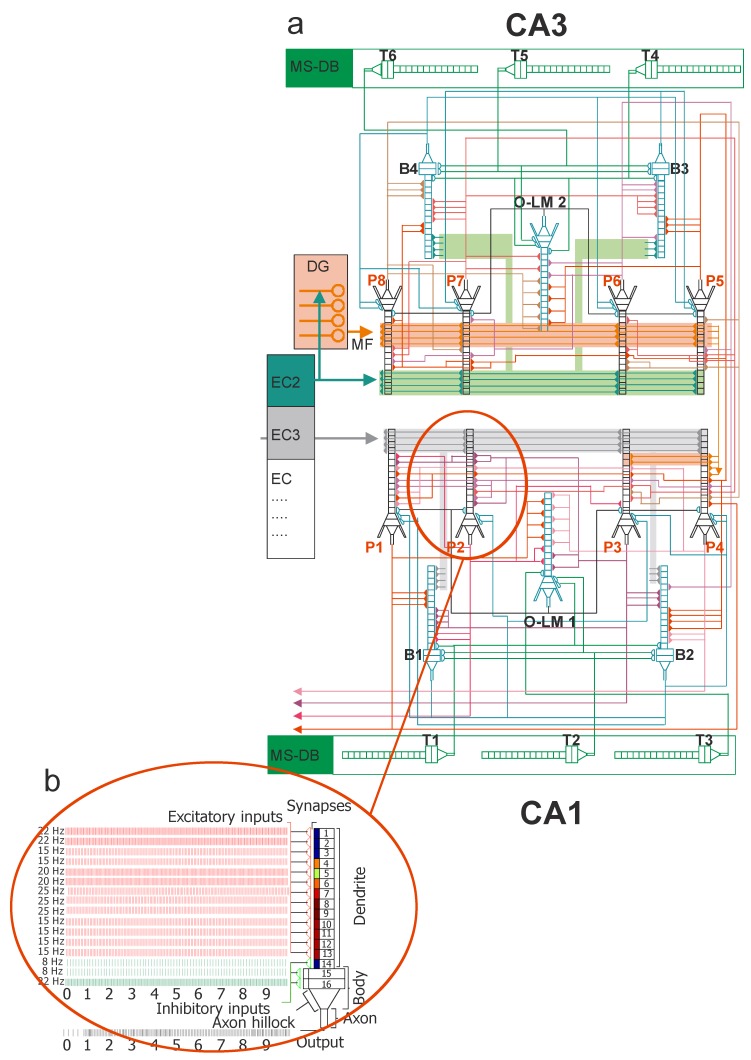
(**a**) The CA3-CA1 hippocampal formation microcircuit. On the top is the CA3 region and CA1 is at the bottom. Major cell types and their connectivity: pyramidal (P1–P8), basket (B1–B4), OL-M (1–2) cells, mossy cell (MC). T1–T6 represent GABAergic cells in the medial septum-diagonal band (MS-DB) region. Every CA1, CA3 pyramidal cells, basket and O-LM cells consist of 16 compartments in which each dendrite has an excitatory or inhibitory synapse (example–pyramidal cell on the left at bottom). (**b**) Example–pyramidal cell. Red excitatory, green inhibitory inputs. Pyramidal cell model configuration with color filled postsynaptic regions according to values of LTP at the end of simulation.

**Figure 2:**
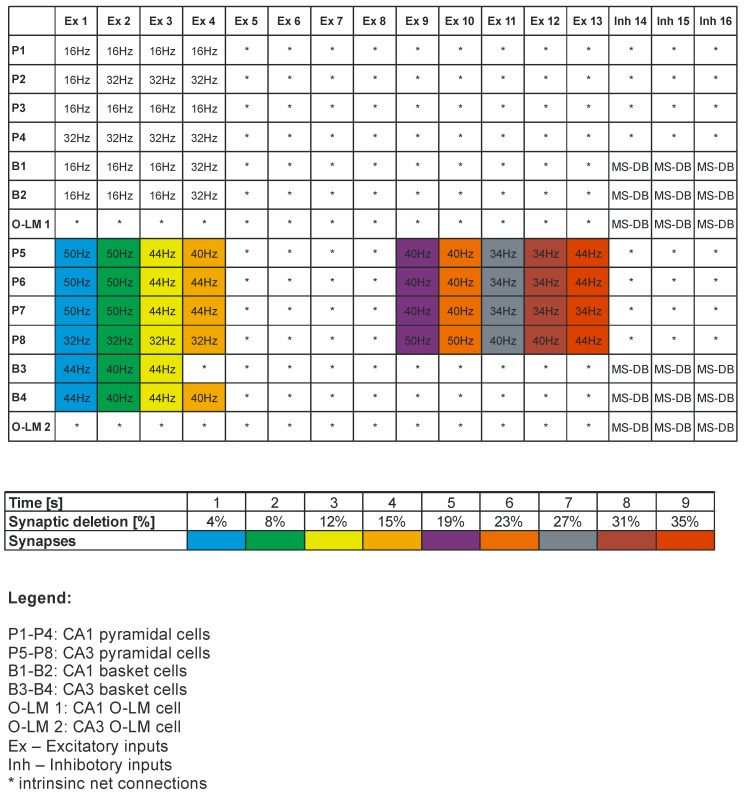
On Pyramidal cells from CA3 and CA1 regions we have together 8*13 = 104 (100%) excitatory synapses. Color marker the deleted synapses during simulation. The deletions were made only in CA3 microcircuit.

The simulated microcircuit model of CA3-CA1 network is presented in Figure 1. This model consists of 4 CA3 pyramidal cells (PCs), 2 CA3 basket cells (BCs), 1 CA3 oriens-lacunosum/moleculare cell (O-LM cell), 4 CA1 PCs, 2 CA1 BCs, and 1 CA1 O-LM cell. The simplified morphology of neural cells includes a soma, a portion of the axon and both apical and basal dendrites. All properties of neural cells used in the experiment were based on features reported in the literature [4,26,27,28,29,30]. Modeling of AD consisted of turning off one connection after the other from EC2 to the CA3 pyramidal neurons, to inhibitory interneurons (basket cells) as well as the mossy fibers synapses on CA 3 pyramidal cells (Supplementary Materials: Video AD: CA3-CA1 network simulation of Alzheimer disease-pathology model, Video healthy-control model: CA3-CA1 network simulation of healthy). For further details, see Figure 2. 

### 2.1. CA3 Cells

Every CA3 pyramidal cell, CA3 basket cell and CA3 O-LM cell are constructed with compartments and every dendrite has a synapse, which is either excitatory or inhibitory. Excitatory inputs in stratum lucid dendrites are received from CA3 pyramidal cells, EC2 and dentate gyrus (DG). Excitatory connections are received by their distal dendrites from EC2 and by medium dendrites from DG granule cells’ mossy fibers. Inhibitory impulses come from connections in the medial septum of their somas. Each O-LM cell receives excitatory and inhibitory connections. The first ones are received from active CA3 pyramidal cells in their basal dendrites, and the second ones from the medial septum. The mathematical formalism of the models was based on our previous studies [31,32,33]. 

### 2.2. CA1 Cells

Every CA1 pyramidal cell, CA1 basket cell and CA1 O-LM cell is constructed with 16 compartments in which each dendrite has an excitatory or inhibitory synapse. Each CA1 cell receives somatic synaptic inhibition from CA1 basket cells, proximal excitation from CA1 pyramidal cells, mid-dendritic excitation from mossy fibers (MF) and distal apical excitation from EC layer 3 (EC3) [1,2]. Each basket cell receives somatic synaptic inhibition from the medial septum (Theta oscillations) and adjacent basket cells in their soma. Excitatory connections are provided to their distal dendrites from EC3 and to medium dendrites from both CA1 pyramidal cells and CA3 Schaffer collaterals. Each O-LM cell receives excitatory and inhibitory connections. The first ones are received from active CA1 cells in their basal dendrites, and the second ones are received from the medial septum (Theta oscillations).

### 2.3. Model Inputs

Inputs from the CA3-CA1 model came from MS and layers 2 and 3, as proved by Witter [34]. There were two separate populations of cells with theta-modulated strength, and all inputs were modeled as firing. Every input was modeled as the firing of one hundred entorhinal cortical cells at a theta frequency of 8 Hz. The CA3 PCs and CA3 BCs acquired input in the apical dendrites from EC2, and CA1 PCs and CA3 BCs from EC3 in their apical lacunosum moleculare dendrites (LM) [4]. Inputs acquired from layers 2 and 3 were modulated as opposites, when one input was strong, the other was weak, and vice versa [4]. All initial parameter microcircuit models of the CA1-CA3 network are presented in Table 1 and Table 2 where low significant weight (LSW)–is the weight of the highest distal dendrite input. The typical time course of excitatory or inhibitory postsynaptic potentials: is EPSPd or IPSPd.

### 2.4. Synaptic Properties

For this model, several synapses were considered, including AMPA, NMDA and GABA-A. AMPA synapses were observed in CA1 and CA3 strata LM (EC2 and EC3 connections), distal two-thirds of the molecular layer (EC2 connections), CA3 lucidum (DG-GC connections) and CA1 radiatum (CA3-PC connections). The NMDA synapses existed in CA3 lucidum (DG-GC connections) and CA1 radiatum (CA3-PC connections). The GABA-A synapses were inherent in almost every CA1 and CA3 strata and layers [19]. There are glutamate receptors for excitatory inputs: AMPA - E (k, i), NMDA - M (k, i). The GABA receptors are for inhibitory inputs: I (k, i), where k is the number of dendrite compartments and i the number of area- register tables. Each area simulates 0.5 ms of real time and is primitively filled with the resting potential from value ReP *=* −80 mV. The long-term potentiation (LTP) induction occurs only if depolarization of the postsynaptic region in NMDA channels, is sufficient and then the weight of this synapse is increased.

### 2.5. Memory (LTP)

**Figure 3:**
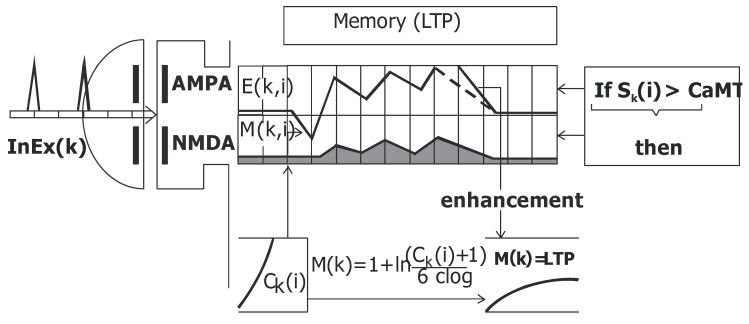
Organization of program functions loops for calculation of the LTP value.

Each synapse is checked and after a detection of an action potential the values of the table of a certain shift register are changed by synaptic function according to the typical time course of postsynaptic excitatory (EPSP) or inhibitory (IPSP) potentials. For every 0.5 ms of the simulated time, the actual values of all registers are checked and their weighted sums are compared with related thresholds. There is a positive correlation between the weight and a related synapse’s proximity to the cell body. The axon provides the output signal to the compartments of the dendrites of the destination pyramidal cell. When an action-potential is generated, the registers are reset to the value of resting potential and then the activity of all compartments is inhibited for a 1.5 ms period of refraction. Yet we should also have a memory related to each input and working according to LTP rule. In each state-updating cycle the program calculates (based on actual values in the registers and appropriate weights) an accumulated local potential for postsynaptic region of each input synapse. If, at moment of arrival an action potential at a particular input, the postsynaptic potential is beyond the value of the local threshold (ca. 70mV), the second register (M-which simulates the rise of calcium ion content inside the cell) change values of their data-boxes. The enhanced strength remains for a time calculated as power function of the charge, which diminishes according to the forgetting coefficient (FQ) shown in Figure 3. The formal expression can be written as:

(1) If Sk(i) > CaMT then (2)
  (CaMT = −68mV threshold for remove of Mg ion block)
(2) Time of duration of memory:
  $$C_{k}\left(i+1\right){=C}_{k}\left(i\right)+\exp\left(10\left(M_{k,0}\left(i\right)-\mathrm{ReP}\right)\right)-1$$
  $$\mathrm{if}C_{k}\left(i+1\right)>\mathrm{FQ}\;\mathrm{then}C_{k}\left(i+1\right){珻}_{k}\left(i+1\right)-FQ$$
  Mk,0- actual value of synaptic function for EPSP (NMDA) in register M.
  ReP = −80mV (resting potential).
  FQ-forgetting coefficient not below 1.
(3) Memory (LTP)
  $$M(k)=1+ln\frac{\left(C_{k}\left(i\right)+1\right)}{6clog}$$
  Ck(i) time of memory for compartment (k), clog parameter = 2.3026

Thus, we have always the rapid weight increase and very slow as compare to STDP algorithm decrease of synaptic weights. And the FQ parameter could be independently matched to each neuron before network simulation.

### 2.6. Correlation Dimension, Shannon Entropy, and Embedding Dimension

Nonlinear analysis of the results of control model simulation in pathologies allowed for the reconstruction of the phase space as a method describing the complexity of the dynamic system [35]. Reconstruction of the attractor used the time delay method [36,37]. In contrast, the method of the false nearest neighbors selected a minimum dimension of deposition of one-dimensional time series of simulation results of neural networks [38].

### 2.7. Statistical Methods

The statistical analyses have been performed using TIBCO Software Inc. (2017). Statistica (data analysis software system), version 13. http://statistica.io. We also present results from Visual Recurrence Analysis (VRA) of Kononov as selected from various methods for recurrence plots analysis [39]. Statistical significance of differences between two groups was processed with the t-Student test or U Mann-Whitney test. The significance of difference between more than two groups were assessed with F test or Kruskal-Wallis H test. In the case of statistically significant differences between two groups, post-hoc tests were utilized. Chi-squared tests for independence were used for qualitative variables. In order to determine dependence, strength and direction between variables, correlation analysis was used by determining the Pearson (and Spearman’s rank-order) correlation coefficients. In all calculations, the statistical significance level of p < 0.05 has been used.

## 3. Results

Cells in CA1 and CA3 rhythmically phase their population activities at opposite half-cycles of theta, which seems to be crucial in the encoding and retrieval of memories [33,34,40,41,42]. In the theta phase separation of these processes in half-cycles of theta, a basic role is played by dendritic, somatic and axsonic inhibition [31,32,33,43]. 

**Figure 4:**
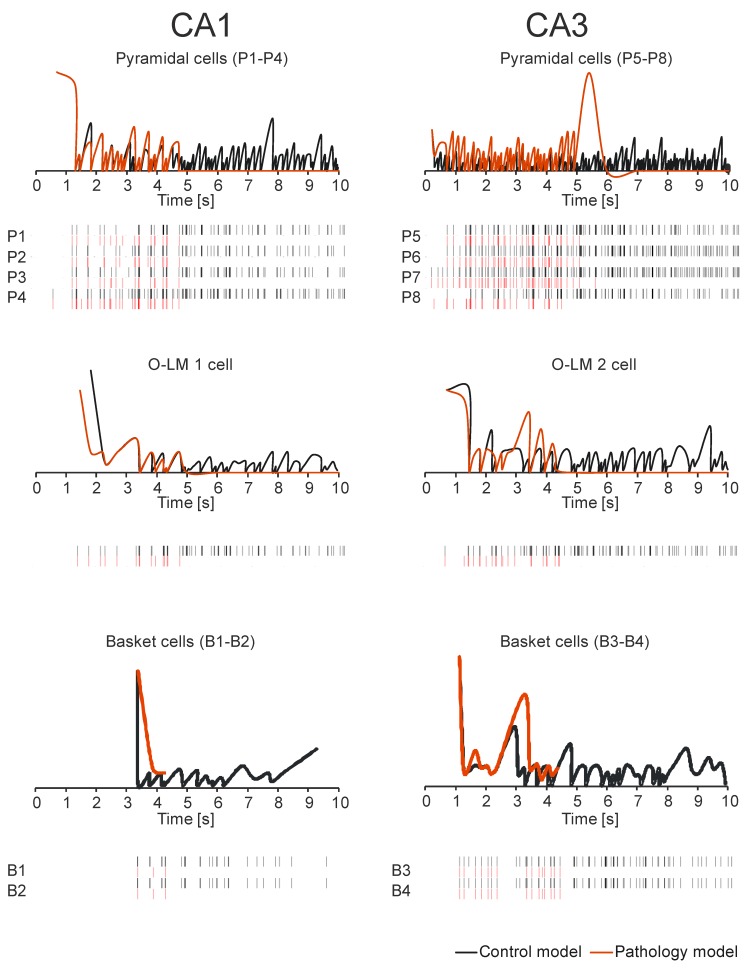
Ten second real-time simulation of CA3-CA1 network control model and pathology model. The time course of Interspike Intervals (ISI) and output spikes trains for main cells of CA3-CA1 formations (top-pyramidal cells (P1-P8), medium-OL-M cell (1 and 2) and bottom basket cells (B1-B4)).

In an experiment conducted on a normal hippocampus after 10 seconds of stimulation, the received spike values for CA1 pyramidal cells were from 72 to 135 (mean 92.25, SD 28.999); however, the received spike values for CA3 pyramidal cells were from 260 to 313 (mean 178.50, SD 56.736) Figure 2 and Figure 3. In an experiment carried out on an AD hippocampus, after 10 seconds of stimulation the received spike values for CA1 pyramidal cells were from 18 to 38 (mean 24.00, SD 9.381); however, the received spike values for CA3 pyramidal cells were from 32 to 56 (mean 42.75, SD 10.308). There were statistically higher spike values of both CA1 and CA3 pyramidal cells in the normal hippocampus than in AD (p = 0.0042 for CA1 and p = 0.0033 for CA3) (Figure 4). The average frequency of normal CA1 pyramidal cells was 64.95 Hz (SD 70.04 Hz), however in AD the average frequency was 36.31 Hz (SD 57.419 Hz). The conclusion which can be drawn is that in the normal hippocampus, the frequency of CA1 pyramidal cells is statistically higher (p = 0.0002) (Figure 4). After a 10-second stimulation of normal pyramidal CA1 cells, the final received frequency was 21.79 Hz (SD 15.833 Hz); however, in AD it was 3.23 Hz (SD 1.190 Hz). 

**Figure 5:**
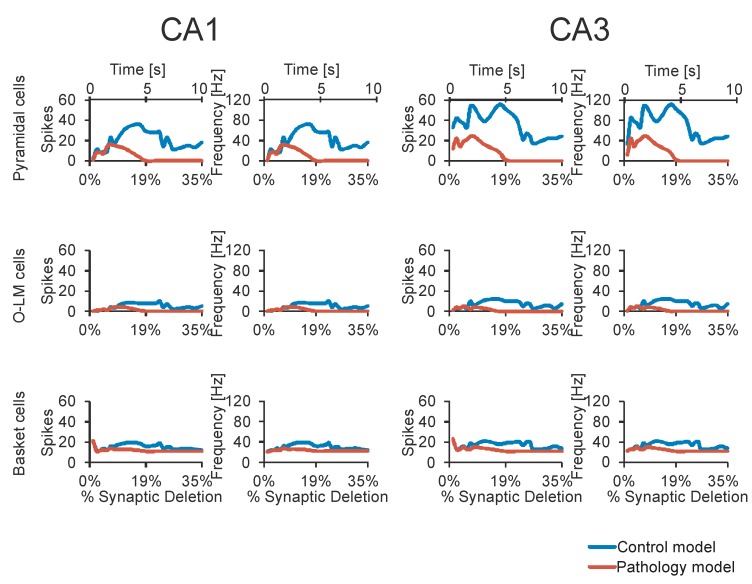
Relationship between number of spikes, frequency and synaptic deletion control and pathology models (pyramidal (P1-P8), OL-M (1 and 2) and basket (B1-B4) cells). The CA1 region is on the **left** and is the CA3 formation is on the **right**.

The difference between the final frequency observed in the normal and AD hippocampus was almost 7, which proves the existence of a relevant decrease in CA1 pyramidal cell activity. The average frequency of normal CA3 pyramidal cells was 69.91 Hz (SD 62.876 Hz); however, in AD the average frequency was 32.69 Hz (SD 43.173 Hz). The conclusion is that in the normal hippocampus the frequency of CA1 pyramidal cells is statistically higher (p = 0.0001) (Figure 5).

After a 10-second stimulation of normal pyramidal CA3 cells, the final received frequency was 28.95 Hz (SD 18.483 Hz); however, in AD it was 4.74 Hz (SD 2.081 Hz). The difference between the final frequency observed in normal and AD hippocampus was almost 6, which proves the existence of a relevant decrease in CA3 pyramidal cell activity. 

The comparison of spike values and frequency dependence from a synaptic deletion in CA1 and CA3 is presented in Figure 3. In AD high and statistically significant correlations between spike values and synaptic deletion in both CA1 and CA3 cells (correlation coefficient from −0.64 to 0.85, p < 0.05) were received. The progression of AD was consolidated with the decrease of spike values and frequency, whereas the results received after the stimulation of normal hippocampus were not statistically significant. The CA1 basket cells were the only group of observed cells without a high correlation between frequency and synaptic deletion (correlation coefficient from −0.64 to 0.82, p < 0.05) (Figure 5). Empirical research enabled us to reconstruct the expanse with the delayed method.

**Figure 6:**
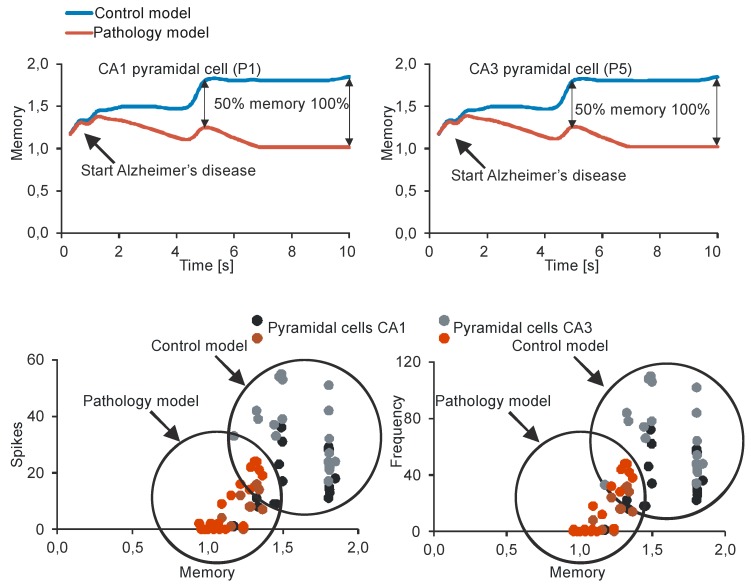
On the **top**: relationship between memory (pyramidal cells (P1 and P5)) and synaptic deletion in control and pathology models CA3 and CA1 formations. On the **bottom**: relationship between number of spikes and memory for control and pathology models. Filled circles for control and pathology models group output simulation train.

The relationships between memory and synaptic deletion healthy and AD CA3 and CA1 formations are presented in Figure 6. There were high statistically correlations between memory and synaptic deletion in both CA1 and CA3 cells. Furthermore, there were correlations between memory and number of spikes in both CA1 and CA3 pyramidal cells.

**Figure 7:**
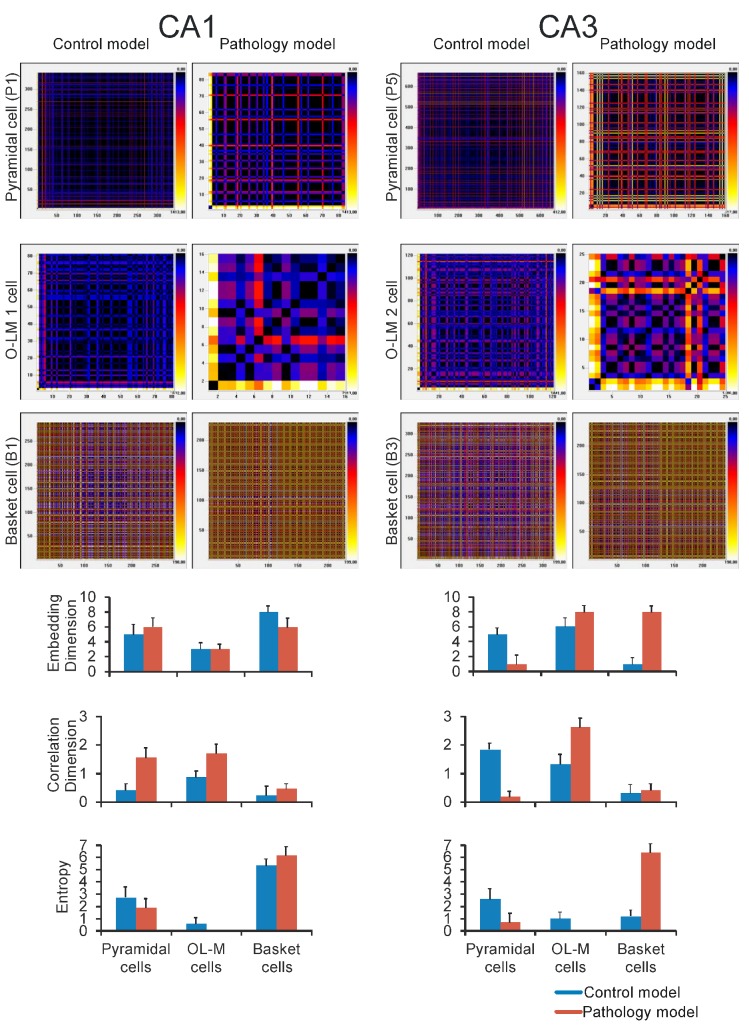
Nonlinear analysis of simulations CA1 region (**left**) and CA3 (**right**). On the top recurrence plots with visual recurrence analysis (VRA), which shows all Euclidean distances between data points from the 3-dimensional phase space for all cell types (pyramidal, OL-M and basket). On the bottom: comparison of control and pathology models embedding dimension, correlation dimension, entropy for pyramidal (P1 and P5), OL-M (1 and 2) and basket cells (B1 and B3).

The time of the delay was estimated according to the analysis of the autocorrelation function. A range of methods can be used to show regular and/or chaotic behavior of the CA3-CA1 model and multiple neural spike train data analysis. In our paper we present the visual recurrence analysis (VRA) method, which was expanded upon by Eugene Kononov and selected from a variety of methods. The results are presented in Figure 7. Moreover, Figure 7 compares embedding dimension, correlation dimension and entropy between healthy and AD hippocampus. There were no statistical differences in the embedding dimension between healthy and AD (p = 0.6650) for pyramidal cells of the CA1 region and CA3 network (p = 1.00) (Figure 5). There were no statistical differences in the correlation dimension between healthy and AD (p = 0.6650) for pyramidal cells of the CA1 region and CA3 network (p = 0.8852). A similar outcome was achieved for entropy pyramidal cells of CA1 (p = 0.1124). However, the entropy of pyramidal cells of the CA3 network for healthy seemed to be significantly higher (p = 0.0304) than AD (Figure 7).

## 4. Discussion

AD affects different parts of the brain when it comes to the late stage of the disease. The first changes are visible in the mesial temporal lobe and comprise hippocampal formation, which seems to be of particular significance in memory function process impaired by AD [1]. There are several models describing memory properties in various ways, which lead to a hypothesis that the implementation of multiple models may result in ameliorated performance [44]. The attractor neural model is based on the assumption that memory corresponds to a constant spatiotemporal standard of activated neurons and simulates some memory features such as error correction and pattern completion [1,2,3]. Stable states correspond to a variety of memories and might be termed as attractor states or fixed-point attractors. In any network containing N neurons all activation states are expressed by a point in an N-dimensional feature space. Assuming that the activation state of any neuron could be expressed by one or two states (0 or 1), the total number of eventual memory states is 2-N. The network gravitates to the attractor state if only activated in an initial state that is similar to the attractor’s. The final state of a network always belongs to one of the attractor states; however, the selection of direct attractor states depends on the initial state. This model focuses on the true memory even if incorrect memory information is prevalent.

In this model all varieties of memory patterns are stored as weights of synaptic connections [1,2]. Although error correction and pattern recognition are simulated properly, there appears to be a valid drawback with respect to encoding a new input, which is also perceived as an incorrect pattern [1]. There is an assumption that hippocampal processing switches to various models during the implementation of a variety of functions and this model is an extension of the attractor neural model. In experimental studies, capital effort is required for memory recall compared with recognition, which is mentioned by Hasselmo and McClelland [2]. 

In AD, cell death is limited to 10% of the neuronal population, although there is no warranted cognitive deficit. There is a conclusion based on conducted experiments that a 50% decrease in the number of synaptic connections is the main reliable factor of cognitive deficiency, which is called synaptic deletion [1]. The successful compensating mechanisms of the brain include strengthening the remaining synaptic connections, termed synaptic compensations. Although in more advanced stages of disease neuromodulation it is insufficient to overcome the loss of synaptic connections that lead to greater cognitive deficiency, the synaptic deletion and compensation model is based on those observations [45,46,47,48]. According to this model, it has been proved that in a Hopfield artificial neural networks (ANN) architecture, the loss of synaptic connections is the reason for the decline of memory and disfigurement of learned patterns. However, runaway synaptic modification is another phenomenon perceived in an associative network [49,50,51]. In memory associative models, storage of one memory is connected with storage of related memories and can result in a visible increase in the number of network associations. This phenomenon is believed to be the main cause of the pathological increase of synaptic connection strength and neuronal activity, which result in excitotoxicity. 

As for currently available simulators of life-like neural circuits, they allow us to simulate the circuits built of thousands of cells [52,53], but often tend to go deeply into detailed biophysics and biochemistry of a single neuron; so, in case of simulation of large neural networks, the complexity of the neuron’s mathematical description requires hardly available computational power e.g. Hines [54]; Bower & Beeman [55], Traub et al. [56]. And for practical reasons, the simulation algorithms for network and rule should be as simple as possible for minimal acceptable biological plausibility. As we agree to avoid unnecessarily complications, the STDP algorithm remains useful for synaptic weights changes. It could be regarded as a major evolution of the old Hebb rule and needs precise timing of postsynaptic spikes. However, this is not always true in biology; on distal dendrites the weight changes could occur even without any related postsynaptic activity. Thus, the presented LTP related algorithm can solve this problem; it works on the dendrite level, independently for each compartment and in precise concomitance with the history of all input patterns. 

The set of functions employed in our model should be subject to further implementation in hardware, for which analog memories and field transistors witch floating gate technology are being considered [57,58]. However, we do already have some implementations of STDP synapses in silicon, even nanotechnology projects [59,60]. For implementation of the LTP synapse, the problem of very slow diminishing process that was previously injected to a floating gate charge remains to be solved, and there must be precise regulation possibility of such a process.

There are special mechanisms present in the brain responsible for the reduction of interference caused by excitotoxicity, which was proved by using the runaway synaptic modification model. In this theory the excitatory and inhibitory cholinergic neuromodulation seems to be the main factor in adjusting switching from one mode to another. Runaway synaptic modulation in normal states is prevented by neuromodulation; however, in pathological states, including AD, it is inescapable [47,49,52]. 

Neural models presenting the process of memory are a means to describing mechanisms underlying AD and its progression, although current knowledge is evolving and a full explanation is still unavailable.

## Figures and Tables

**Table 1:** Initial parameters microcircuit model of CA1 network.

CA1 Cells	LSW	EPSPd [mV]	IPSPd [mV]
Pyramidal cell (P1)	0.2	4.5	−6
Pyramidal cell (P2)	0.2	4.5	−6
Pyramidal cell (P3)	0.2	4.5	−6
Pyramidal cell (P4)	0.2	4.5	−6
Basket cell (B1)	1	4	−4.5
Basket cell (B2)	1	4	−5.5
O-LM 1 cell	0.6	4	−4

**Table 2:** Initial parameters microcircuit model of CA3 network.

CA3 Cells	LSW	EPSPd [mV]	IPSPd [mV]
Pyramidal cell (P5)	0.6	4.5	−6
Pyramidal cell (P6)	0.6	4.5	−6
Pyramidal cell (P7)	0.6	4.5	−6
Pyramidal cell (P8)	0.6	4.5	−6
Basket cell (B3)	1	4	−4.5
Basket cell (B4)	1	4	−5.5
O-LM 2 cell	0.6	4	−4

## Supplementary Materials

Video AD: CA3-CA1 network simulation of Alzheimer disease-pathology model, Video healthy-control model: CA3-CA1 network simulation of healthy.
